# Leadership in radiology in the era of technological advancements and artificial intelligence

**DOI:** 10.1007/s00330-025-11745-4

**Published:** 2025-06-27

**Authors:** Barbara D. Wichtmann, Daniel Paech, Oleg S. Pianykh, Susie Y. Huang, Steven E. Seltzer, James Brink, Fiona M. Fennessy

**Affiliations:** 1 Department of Radiology, Mass General Brigham, Boston, MA USA; 2 Clinic of Neuroradiology, University Hospital Bonn, Bonn, Germany; 3 German Center for Neurodegenerative Diseases (DZNE), Bonn, Germany

**Keywords:** Radiology, Leadership, Artificial Intelligence, Governance, Workflow

## Abstract

**Abstract:**

Radiology has evolved from the pioneering days of X-ray imaging to a field rich in advanced technologies on the cusp of a transformative future driven by artificial intelligence (AI). As imaging workloads grow in volume and complexity, and economic as well as environmental pressures intensify, visionary leadership is needed to navigate the unprecedented challenges and opportunities ahead. Leveraging its strengths in automation, accuracy and objectivity, AI will profoundly impact all aspects of radiology practice—from workflow management, to imaging, diagnostics, reporting and data-driven analytics—freeing radiologists to focus on value-driven tasks that improve patient care. However, successful AI integration requires strong leadership and robust governance structures to oversee algorithm evaluation, deployment, and ongoing maintenance, steering the transition from static to continuous learning systems. The vision of a “diagnostic cockpit” that integrates multidimensional data for quantitative precision diagnoses depends on visionary leadership that fosters innovation and interdisciplinary collaboration. Through administrative automation, precision medicine, and predictive analytics, AI can enhance operational efficiency, reduce administrative burden, and optimize resource allocation, leading to substantial cost reductions. Leaders need to understand not only the technical aspects but also the complex human, administrative, and organizational challenges of AI’s implementation. Establishing sound governance and organizational frameworks will be essential to ensure ethical compliance and appropriate oversight of AI algorithms. As radiology advances toward this AI-driven future, leaders must cultivate an environment where technology enhances rather than replaces human skills, upholding an unwavering commitment to human-centered care. Their vision will define radiology’s pioneering role in AI-enabled healthcare transformation.

**Key Points:**

***Question*** *Artificial intelligence (AI) will transform radiology, improving workflow efficiency, reducing administrative burden, and optimizing resource allocation to meet imaging workloads’ increasing complexity and volume.*

***Findings*** *Strong leadership and governance ensure ethical deployment of AI, steering the transition from static to continuous learning systems while fostering interdisciplinary innovation and collaboration.*

***Clinical relevance*** *Visionary leaders must harness AI to enhance, rather than replace, the role of professionals in radiology, advancing human-centered care while pioneering healthcare transformation*.

**Graphical Abstract:**

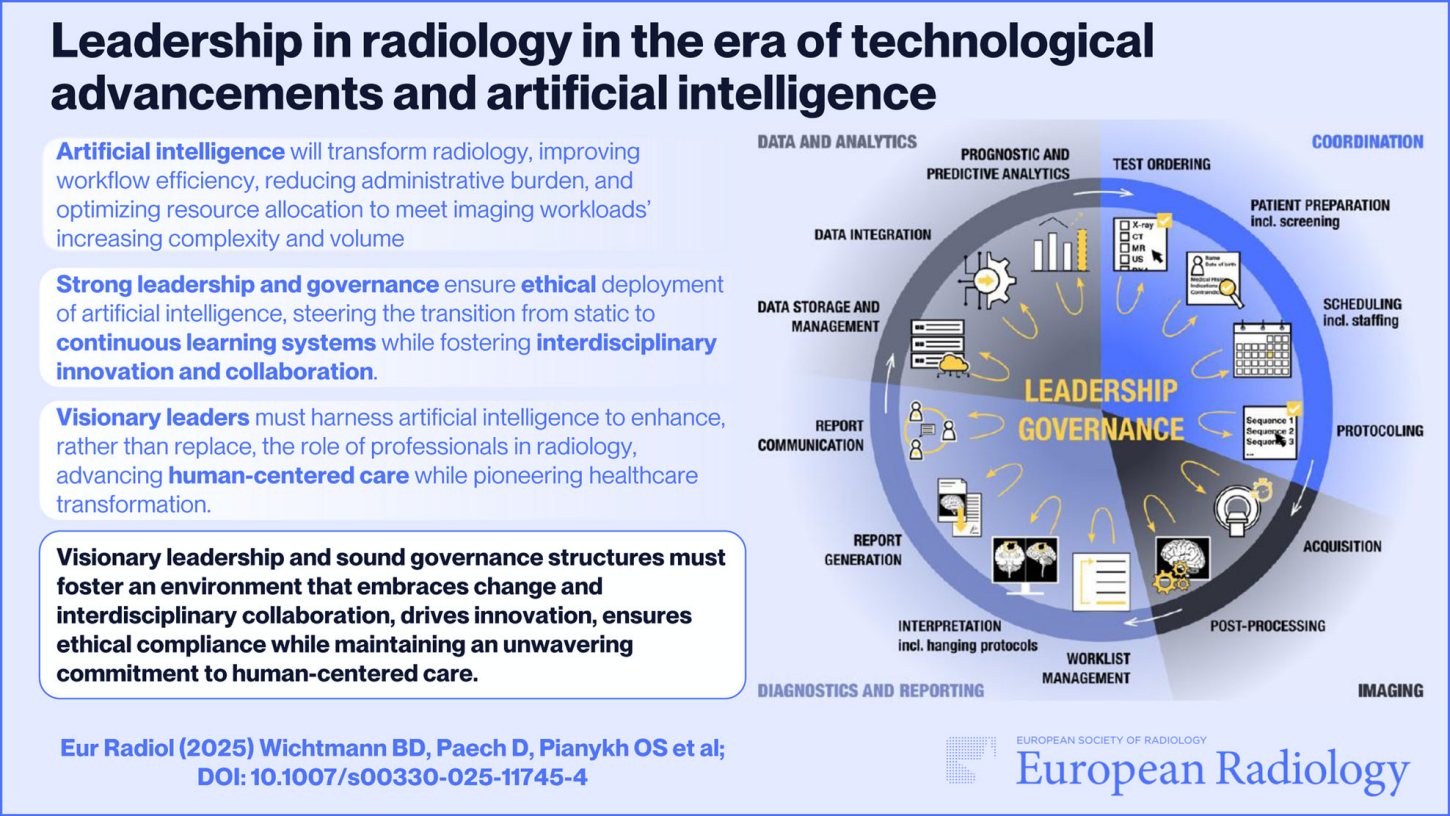

Radiology has been the birthplace of many disruptive technologies. With recent technological advancements and the increasing emergence of artificial intelligence (AI), our specialty stands at the brink of a new frontier, requiring visionary leadership to navigate the unprecedented opportunities and challenges ahead and steer radiology through this transformative journey.

From the early days of X-ray imaging, radiology has continually evolved, adopting cross-sectional anatomic imaging with computed tomography (CT) and magnetic resonance imaging (MRI) to various forms of functional and molecular imaging [1, 2]. The first CT images were computed in 1971, already relying on advanced mathematical data transforms, which were as novel then as using AI algorithms now [3]. Soon after, radiology pioneered the development of the first interoperable standards to transmit, store, retrieve, print, process, and display medical imaging information (such as DICOM), enabling global sharing and interpretation of medical imaging data [4].

Now an integral part of modern medicine, the extensive use and associated cost of imaging studies and procedures, in addition to issues with the environmental sustainability of advanced technologies, pose major challenges to radiology [5]. The need for 24/7 coverage, coupled with a rising workload, growing case volume and complexity, and an increasing number of images per examination, has led to a widespread burnout crisis amid an acute shortage of radiology professionals [6, 7]. Leaders can play a key role in exacerbating or alleviating these challenges through their operational and financial decisions [8].

Just as digitalization has enabled us to function at a higher level, AI will profoundly impact all aspects of radiology practice, from workflow management to enhancing diagnostic accuracy and efficiency to developing prognostic and predictive image-based markers (Fig. 1) [1, 9]. The delegation of routine tasks could enable radiologists to focus on higher-value and creative tasks, and on more meaningful interpersonal interactions.

**Fig. 1:**
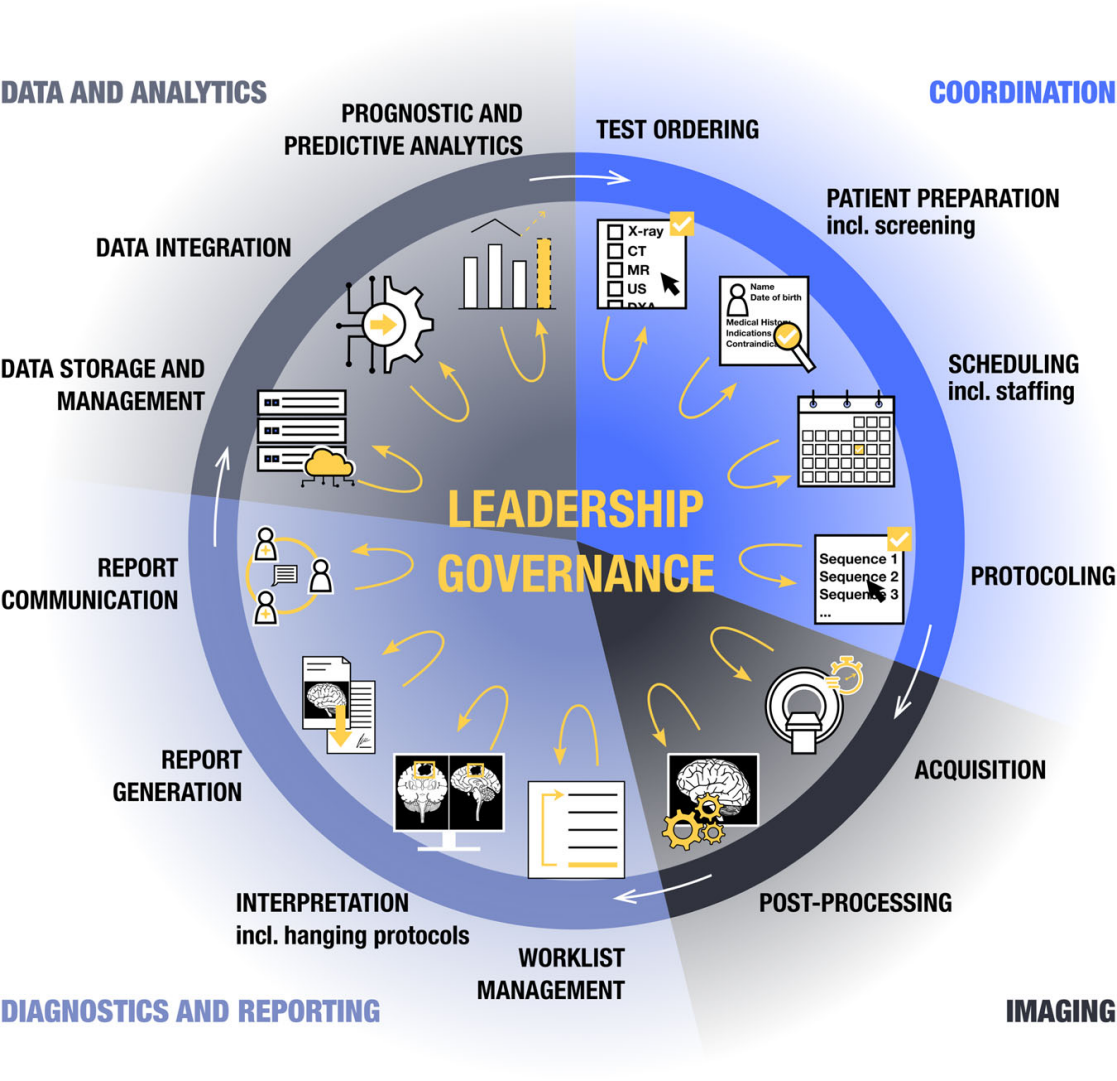
Leveraging its strengths in automation, accuracy and objectivity, AI can optimize every step of the diagnostic imaging workflow, from coordination to imaging, diagnostics, reporting and data-driven analytics. Leadership and governance structures are central to the continuous assessment and adaptation of AI solutions, steering the transition from static to continuous learning systems. A strong leadership and governance framework will be needed to ensure ethical oversight and interdisciplinary collaboration as these systems advance and gain new insights from evolving environments. This balanced approach supports the sustainable integration of AI across the imaging enterprise, from operational efficiency to diagnostic accuracy and predictive analytics, enabling more efficient, precise and human-centered care

While the potential of AI in radiology is significant, the fragmented landscape of AI solutions complicates seamless integration into existing clinical workflows, demanding extensive interoperability efforts [10]. To date, relatively few AI applications have achieved widespread adoption in clinical practice [11]. Successful implementations have emerged in select areas, including AI-assisted stroke triage and infarct segmentation demonstrating improvements in diagnostic speed and accuracy [12, 13], population-based mammography screening reducing workload while improving cancer detection and specificity [14, 15], or AI-driven MRI acceleration shortening acquisition times without compromising image quality [16].

Identifying optimal AI applications within the diverse radiological workflows remains complex, requiring robust governance mechanisms for careful selection, validation, and monitoring [17]. Financial considerations, such as substantial initial investment and ongoing maintenance costs, can be barriers for widespread implementation, especially in resource-limited settings [18]. Moreover, issues of human-machine interaction must be carefully addressed, including risks of overreliance on AI outputs or, conversely, under-utilization due to distrust or lack of proper understanding by medical staff [19, 20]. Before AI systems can be fully integrated into daily routines, they must evolve from static to continuously learning systems [21]. Furthermore, the regulatory landscape, including recent frameworks such as the European AI Act, imposes stringent compliance requirements that AI systems must fulfill to ensure patient safety, transparency, and accountability [22]. The implementation of clinical AI in radiology requires strong leadership and robust governance frameworks to oversee not only algorithm evaluation, selection and deployment, but also ongoing maintenance through transparent performance monitoring, automated quality controls, and regular retraining [17, 21].

Today, medical technologies have evolved to such an extent that digital data from various clinical fields, including radiology, pathology, genomics, and metabolomics, can be rationalized and integrated to provide quantitative precision diagnoses powered by AI [23]. We can envision the creation of a “diagnostic cockpit” that allows us to harness multidimensional data to generate comprehensive insights and personalized treatment strategies [23]. Realizing this vision relies on interdisciplinary collaboration and visionary leadership from within the medical imaging community that fosters innovation [17, 23]. Key steps will include the evaluation and clinical validation of AI systems, a transformative process that must be primarily driven and advanced by academic radiology, working in close collaboration with private practices that handle a large portion of patient care.

By leveraging AI while maintaining a human-centered approach, we can amplify our impact, optimize clinical outcomes, and elevate the experience for both the patients we care for and ourselves [23]. Leaders need to understand not only the technical aspects of AI, but also the complex human, administrative, and organizational challenges associated with its implementation [17]. A key challenge will be to find the right balance between leveraging the potential of AI whilst ensuring that AI enhances, rather than replaces, human skills. Human qualities such as creativity, empathy, and emotional intelligence will remain crucial in medical care [24]. By relieving radiology professionals of lower-value routine tasks, AI holds the potential to reduce burnout while preserving or potentially strengthening the role of radiologists in patient care. Just as can be seen in dating and relationship apps, AI may also offer a way to reshape recruitment practices and facilitate optimal mentoring pairings [25].

Rising healthcare costs, driven by both advancements in medical technology and inefficiencies in healthcare delivery systems, call for innovative solutions that support technological integration while maintaining or improving the quality of care. Through administrative automation, precision medicine, and predictive analytics, AI can enhance operational efficiency through streamlined processes, reducing administrative burden, and optimizing resource allocation, ultimately leading to substantial cost reductions [26].

Leadership in radiology demands vision and foresight to navigate the opportunities and challenges of advancing technology and to anticipate the integration and impact of AI on the field. Implementing AI initiatives will drive organizational change that needs to be successfully managed and requires significant financial investment, not just in technology, but also in human capital. The next generation of radiologists will depend on leaders who possess a solid understanding of AI concepts and practical experience in their development and integration, aiming to enhance clinical utility and improve patient outcomes [27]. A key objective will be the cultivation of an ecosystem supported by professional societies, industry, and government bodies that enable close collaborations between clinicians and scientists, driving foundational and translational AI research in radiology [28]. A vital leadership quality will be to build consensus and create institution-wide buy-in and investment in AI by building cross-departmental and cross-disciplinary partnerships. To ensure adherence to ethical principles and appropriate supervision of the implementation, maintenance, and monitoring of AI algorithms, establishing sound governance and organizational frameworks will be essential [17, 29]. Additionally, it will be the responsibility of radiology leaders to ensure that innovation thrives by celebrating successes and fostering a culture of continuous learning that embraces diversity and inclusion through tailored curricula, mentoring and coaching initiatives [27, 30]. While the upcoming changes, along with the infrastructure and investments required for the clinical implementation of AI, will undoubtedly present many challenges, we can expect that this transformation will greatly benefit our field and position us as pioneers in expanding medical practice through AI.

Radiology has evolved from the pioneering days of X-ray imaging to a field rich in advanced technologies now standing on the cusp of a transformative AI-driven future. Radiology leaders must ensure that these technological advances enhance, rather than replace, the role of professionals in radiology. They must foster an environment where AI complements human skills and enables us to focus on value-driven tasks that improve patient care and our environment. As we move towards this new frontier, it is imperative for radiology leaders to embrace change, drive innovation, and maintain an unwavering commitment to human-centered care to ensure that the future of radiology remains both technologically advanced and deeply compassionate.

## Abbreviations

AI: Artificial intelligence
CT: Computed tomography
MRI: Magnetic resonance imaging
